# Integrin Signaling in Glioma Pathogenesis: From Biology to Therapy

**DOI:** 10.3390/ijms21030888

**Published:** 2020-01-30

**Authors:** Aleksandra Ellert-Miklaszewska, Katarzyna Poleszak, Maria Pasierbinska, Bozena Kaminska

**Affiliations:** Laboratory of Molecular Neurobiology, Neurobiology Center, Nencki Institute of Experimental Biology of the Polish Academy of Sciences, 02-093 Warsaw, Poland; a.ellert@nencki.edu.pl (A.E.-M.); k.poleszak@nencki.edu.pl (K.P.); m.pasierbinska@nencki.edu.pl (M.P.)

**Keywords:** integrins, extracellular matrix, tumor-stroma cross talk, gliomas, tumor microenvironment, interfering peptides

## Abstract

Integrins are a large family of transmembrane adhesion receptors, which play a key role in interactions of a cell with the surrounding stroma. Integrins are comprised of non-covalently linked α and β chains, which form heterodimeric receptor complexes. The signals from integrin receptors are combined with those originating from growth factor receptors and participate in orchestrating morphological changes of cells, organization of the cytoskeleton, stimulation of cell proliferation and rescuing cells from programmed cell death induced by extracellular matrix (ECM) detachment. Upon binding to specific ligands or ECM components, integrin dimers activate downstream signaling pathways, including focal adhesion kinase, phosphoinositide-3-kinase (PI3K) and AKT kinases, which regulate migration, invasion, proliferation and survival. Expression of specific integrins is upregulated in both tumor cells and stromal cells in a tumor microenvironment. Therefore, integrins became an attractive therapeutic target for many cancers, including the most common primary brain tumors—gliomas. In this review we provide an overview of the involvement of integrin signaling in glioma pathogenesis, formation of the tumor niche and brain tissue infiltration. We will summarize up-to-date therapeutic strategies for gliomas focused on interference with integrin ligand-receptor signaling.

## 1. Introduction

Glioblastoma (GBM; WHO grade IV) is the most common and aggressive, primary brain tumor in adults [1]. This tumor is considered to be one of the deadliest human malignancies, due to diffusive growth impeding complete surgical resection, genetic alterations driving apoptosis resistance, proliferation and invasion, and poor responses to current therapies. GBM is characterized by genetic and cellular tumor heterogeneity, which encompass inter- and intra-tumor diversity of mutational landscape and a variety of cell types in the tumor niche. This specific niche, called the tumor microenvironment (TME) is composed of recruited immune and stromal cells “re-educated” to facilitate tumor growth. TME contains also a rare population of cancer stem-like cells (called glioma initiating cells, GIC) with self-renewal potential and multidrug resistance. All these features contribute to inevitable tumor recurrence and a very poor prognosis for the patients [2,3].

According to the recommendations provided by the World Health Organization (WHO, 2016), brain tumors are diagnosed using both histology and genetic features, including isocitrate dehydrogenase (IDH) mutation analysis [4]. The *IDH*-wild type GBMs account for approximately 90% of diagnosed cases and correspond most frequently to clinically defined primary or *de novo* glioblastoma. A median patient survival rate is 15 months after diagnosis, with a few months prognostic benefit in case of mutation in *IDH* genes. Postoperative radiotherapy with the addition of concurrent and adjuvant chemotherapy with temozolomide (an alkylating agent) is the current standard of care for newly diagnosed GBM [5]. The quality of life of surviving patients is rather poor as they suffer from serious cognitive impairments related to extensive surgery and chemo/radio-therapy. Moreover, there is no effective treatment for progressive or recurrent GBM, which invariably occurs in most cases. Several new therapeutic modalities that showed a good anti-tumor efficacy in other cancers, such as adjuvant therapies with receptor tyrosine kinase inhibitors, anti-angiogenic therapy with a monoclonal antibody blocking vascular endothelial cell growth factor [6] or T-cell based therapies with immune checkpoint inhibitors [7], failed in clinical trials in recurrent GBMs. The presence of the blood-brain barrier (BBB), which limits the brain entry of non-permissive substances, is an additional obstacle in drug selection. Improvement of the treatment modalities for GBM patients remains a paramount challenge for clinicians and researchers.

Integrins belong to a large family of heterodimeric transmembrane adhesion receptors, named after their roles in “integrating” cell function with the surrounding stroma [8]. Upon binding to ligands or extracellular matrix (ECM) components, integrin dimers activate downstream signaling pathways, which regulate cell migration, invasion, proliferation and survival. Integrin-mediated signaling plays important roles in development and tissue homeostasis, and its deregulation is often associated with diseases. Tumor cells and tumor-associated stromal cells frequently upregulate expression of specific integrins, which is associated with an inferior patient outcome [9]. Deregulated integrin signaling is implicated in driving an invasive phenotype of tumor cells and modulation of a tumor microenvironment to support angiogenesis, activate matrix degrading enzymes, stimulate immune cells trafficking, sustain cancer stem cell niches and regulate organotropism of metastatic cancer cells [9,10,11]. A crosstalk between integrins and cytokines or growth factors immobilized within the ECM is crucial for many aspects of tumor progression [12]. As cell surface receptors that play a key role in cancer biology, integrins represent an attractive target for diagnostic and therapeutic applications.

In the present review, we provide an overview of integrin signaling in glioblastoma. We summarize an up to date information regarding an integrin structure, composition and intracellular pathways involved in signal transduction upon receptor activation. Next, we describe deregulation of integrin signaling in gliomas and focus on its molecular and functional consequences in GBM progression. While in many aspects, the role of integrins is similar across various cancers, we discuss specific functions of integrins in GBM pathogenesis in the specific brain environment. We assess examples of therapeutic targeting of integrins, which despite past disappointing outcomes still holds a promise to be effective against GBM.

## 2. Integrin Structure and Signaling

**Figure 1:**
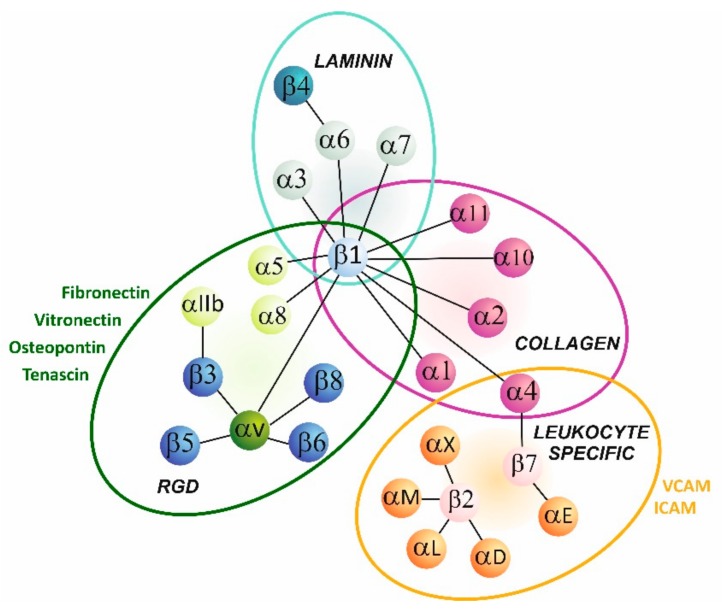
Overview of main integrin heterodimers and their ligands.

The name “integrin” was first proposed by Tamkun et al. in 1986 for a protein complex that linked the extracellular matrix to the actin-based cytoskeleton [13]. Integrins are found in many organisms, from lower eukaryotes such nematodes and sponges to mammals. They are key molecules involved in cell-cell and cell-microenvironment communication. Some integrins bind directly to other transmembrane proteins or soluble ligands, but most of them are receptors for various extracellular matrix components. Integrins act as heterodimers comprised of non-covalently associated α and β subunits. In mammals, there are 18 α and 8 β subunits, forming 24 different heterodimers. Combination of distinctive α and β subunits determines functional specificity of receptors [14]. Based on ligand preferences, integrins can be classified into four groups, i.e., collagen-, laminin- or RGD motif-binding integrins and leucocyte-specific receptors (Figure 1).

Collagen receptors are composed of α1, α2, α10 and α11 subunits forming heterodimers with β1 subunit. In laminin receptors, α3, α6 and α7 subunits pair mostly with β1 [14,15]. Arginine-glycine-aspartic acid (RGD) motif binding integrins comprise a series of αv dimers formed with β1, β3, β5, β6 and β8 subunits, namely αIIbβ3, α5β1 and α8β1. Fibrinogen, fibronectin, tenascin, vitronectin, osteopontin are examples of proteins being ligands containing the RDG motif but their list is far more extensive [16]. Integrins expressed predominantly by leukocytes consist of one of several α subunit counterparts αL, αM, αX, and αD coupled with a β2 subunit. They regulate cell-to-cell interactions in the innate and adaptive immunity. Subunits α4 and α9 belong to another group, referred as leucine-valine-aspartic acid (LVD) binding integrins. These α subunits interact with β1 and β7, and share similar ligands to leucocyte-specific integrins, for example vascular cell adhesion molecules (VCAM) and intracellular adhesion molecules (ICAM) [14,15].

In comparison to other receptors, integrins have a unique function of bidirectional signaling to integrate extra- and intracellular environments. This signaling can occur via two mechanisms referred as “outside-in” and “inside-out”. Outside-in signaling occurs when integrins bind various extracellular ligands and transduce a signal to cytoplasm. Additionally, cytoplasmic proteins can initiate the “inside-out” signaling and modulate an affinity of integrins for their extracellular ligands [17]. Formation of integrin-based adhesion sites generates a series of dynamic events, called mechanotransduction, leading to rapid changes in cellular mechanics as well as to long-term adaptation of a cell to the surrounding microenvironment by releasing biochemical signals [15].

**Figure 2:**
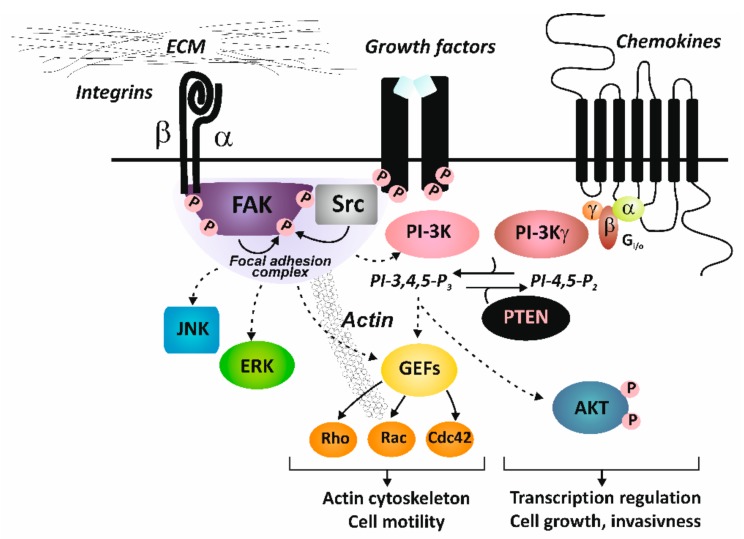
Integrin signaling. Abbreviations: AKT, AKT8 virus oncogene cellular homolog, kinase; ECM, extracellular matrix; ERK, extracellularly activated kinase; FAK, focal adhesion kinase; GEFs, guanine nucleotide exchange factors; JNK, c-Jun N-terminal kinase; PI-3K, phosphoinositide-3- kinase; PTEN, phosphatase and tensin homologue deleted on chromosome 10, tumor suppressor; Src, Rous sarcoma oncogene cellular homolog, kinase.

Integrins lack an intrinsic catalytic activity and extracellular signals are transduced into a cell through activation of integrin-associated proteins and establishment of focal adhesions with non-receptor tyrosine kinases: focal adhesion kinase (FAK) and Src as the key components, or via direct or indirect interactions of integrins with other cell surface proteins (such as tetraspanins or caveolin). Autophosphorylation of FAK generates a high-affinity binding site for the SH2 (Src homology 2) domain of Src. Once bound, Src mediates phosphorylation of additional tyrosine residues of FAK, creating binding sites for other signaling and adaptor molecules [18,19]. Such processes link integrins to downstream signaling effectors, such as the phosphoinositide- 3-kinase (PI3K)/AKT pathway, RAS or small GTPases (Rac1 GTPases) and mitogen-activated protein kinase (MAPK) pathways. Cellular effects of integrin activation lead to cytoskeleton changes and activation of gene transcription to regulate enhanced cell migration, proliferation and survival [8] (Figure 2).

## 3. Deregulation of Integrin Signaling in Gliomas

By linking an intracellular cytoskeleton with an extracellular environment, integrins play key roles in many processes important for embryogenesis, development and formation of tissue architecture in a whole organism. Defects in integrin signaling are associated with a number of pathologies including cancer [9]. While integrins encoding genes are rarely mutated in cancers, deregulation of integrin signaling is frequent and can occur either due to changes in integrin expression or an abnormal increase in expression of their ligands. Moreover, posttranslational modifications, such as glycosylation, sialylation and carbamylation, modify the conformation of integrins leading to an increased ligand affinity and in consequence to altered integrin signaling in tumors [20,21].

**Figure 3:**
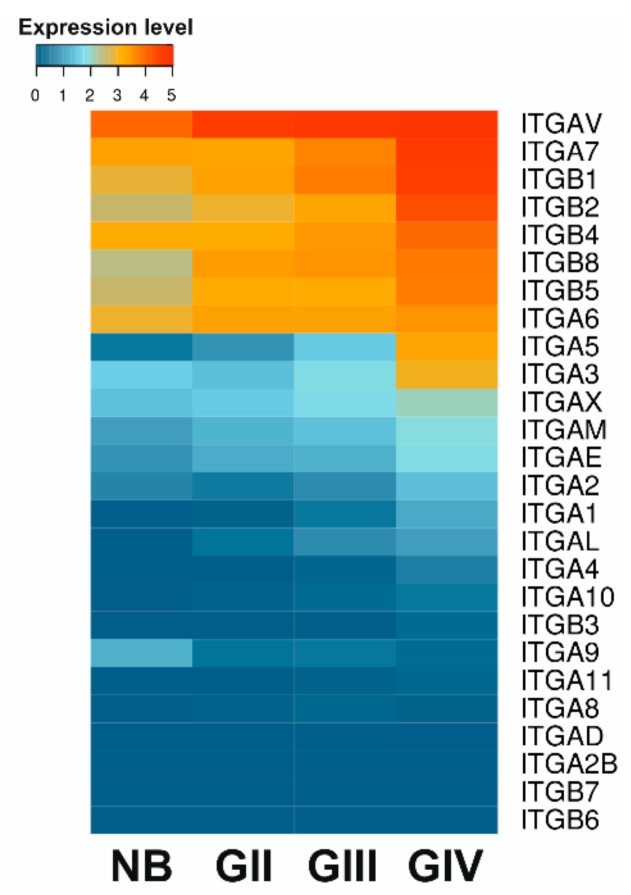
Expression profile of major integrin subunits. Data from 5 normal brain (NB) samples, 248 WHO grade II (GII), 261 WHO grade III (GIII) and 160 glioblastoma (GBM) WHO grade IV (GIV) tumor samples were acquired from TCGA RNAseq repository as FPKM values, quantile normalized and log2 transformed. Mean expression values were compared.

The expression of integrins is upregulated in various cancers, including GBM [9,22]. αvβ3 and αvβ5 were the first integrins identified as differentially expressed in gliomas as compared to normal brain tissues and their expression increased with disease progression. Using transcriptomic RNAseq data available in TCGA (The Cancer Genome Atlas) we compared the expression patterns of major integrin subunits in GBM, lower grade gliomas (LGG, WHO grade II and III) and normal brain samples. Heat-map in the Figure 3 shows upregulation of several integrins in LGG and majority of integrins in GBM samples. Detailed analysis of mRNA expression of integrin subunits in molecular GBM subtypes (as defined by the TCGA network [23]) and their correlation with patient overall survival has also been recently reported [24]. The mesenchymal GBM subtype, which is the most invasive and angiogenic subtype, showed global overexpression of integrins compared to other subtypes, except for integrins β8 and α6, which were overexpressed in the classical subtype [24].

It is worth mentioning that mRNA levels do not always correlate to receptor abundance at a cell surface. This is due to the pairing of integrin receptor subunits that occurs in the endoplasmic reticulum. Only intact heterodimers appear on a cell surface and excessive subunits are degraded [15,25]. Comparative immunohistochemistry staining of integrins in GBM versus control tissues revealed overexpression of α2, α3, α4, α5, α6 and β1, as well as of αvβ3, αvβ5, αvβ8 and α6β1 integrins [26,27], and more recently α7 [28] and α10β1 integrins [29]. Moreover, immunodetection of integrins on tumor sections provided information on localization of these proteins in specific compartments. For example, it was found that αvβ3 is expressed on endothelial cells, αvβ8 is expressed almost exclusively on tumor cells and αvβ5 is expressed in both cell populations [30]. Additional differences in spatial distribution of integrins have demonstrated αvβ5 expression in the tumor core and αvβ3 at its periphery, and enriched α5β1 levels in pre-necrotic and perivascular areas [31,32,33].

Changes in the integrin signaling may originate from variations in the availability of their ligands. Many proteins and proteoglycans forming an ECM are integrin ligands. Remodeling of ECM by tumor cells, via depositing or altering ECM proteins, perturbs proper functions of integrins and promotes tumor invasion. Brain parenchyma has a unique composition as compared to most other organs and is characterized by low density and rigidity [34]. It is composed primarily of hyaluronic acid (HA) bound to proteoglycans of the lectican family, including versican and CNS-specific brevican and neurocan, and some linker proteins [35]. Glioma cells invade the brain parenchyma not randomly, but following preferential routes [36], i.e., along white matter tracts, in perineuronal and perivascular spaces and below the pial margin [35,37]. Such behavior is related to the fact that cells preferably migrate along higher rigidity tracts. A vascular basement membrane has a much higher stiffness than a brain parenchyma and consists of various integrin ligands such as fibronectin, collagen and laminin [38]. Contacts with ECM characterized by an increased stiffness cause among other events upregulation of integrin expression, which further increases the speed of cell migration (for review [39]). Moreover, upon invasion of tumor cells, a brain parenchyma is subjected to substantial remodeling. While degrading the native ECM, GBM cells secrete other ECM proteins, such as tenascin C, fibronectin, vitronectin and collagen, which are present in low quantities in a healthy brain tissue [24], and are known to support invasive phenotype of neoplastic cells in an integrin-dependent manner (reviewed in [35]).

Emerging data continue to uncover new roles for integrins in cancer-relevant pathways [10,11]. Integrins or their transcripts are found in tumor-associated exosomes or other types of small extracellular vesicles (sEV), which are shed by cancer and stromal cells. sEVs act as communication tools within TME and beyond, and transport macromolecules, such as proteins, miRNA, RNA, and DNA, contributing to tumor invasion, neo-angiogenesis, modulation of the immune response, metastatic spread and resistance to treatments [11,40]. Recent proteomic study reported enriched levels of β1 integrin among invasion-related proteins in EVs isolated from GBM compared to those from a low-grade astrocytoma [41]. Corroborative data showed the presence of proteins related to β1 integrin signaling in the sEVs produced by different GBM cell lines and patient-derived stem cells [42]. Moreover, both studies demonstrated that the content of EVs mirrors the phenotypic signature of the respective GBM cells and since tumor-derived sEVs are accessible in biofluids, including the peripheral circulation, they represent novel sources of valuable biomarkers for patient diagnosis and tracking the disease progression [40]. As a validation of this concept, the direct relationship between exosome levels in patient blood and GBM tumor burden has been recently described [43].

## 4. Roles of Integrins and Their Ligands in Shaping A GBM Permissive Microenvironment

### 4.1. Cell Migration and Invasion

**Figure 4:**
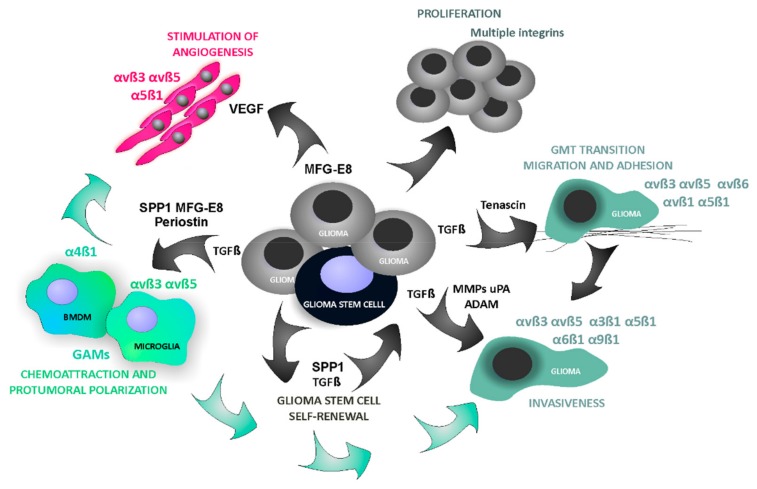
Role of integrins in glioma microenvironment. Abbreviations: GAMs, glioma-associated microglia and macrophages; GMT, glial to mesenchymal transition; MFG-E8, lactadherin; MMPs, metalloproteinases; SPP1, secreted phosphoprotein 1, osteopontin; TGF-β, transforming growth factor; VEGF, vascular endothelial growth factor.

Increasing numbers of experimental and clinical data show that numerous non-neoplastic cells such as macrophages, lymphocytes, neutrophils, mast cells, stromal fibroblasts, pericytes and endothelial cells accumulate within a tumor niche and contribute to tumor growth, progression and resistance to treatments [44,45,46]. This supportive stroma is composed of various cells surrounded by ECM and vasculature creating a unique TME [47,48]. Augmentation of integrin signaling in GBMs affects not only autonomous functions of neoplastic cells, but also the phenotype and behavior of normal stromal cells in the TME. Multiple roles of integrins in various processes related to the pathobiology of GBM are summarized in the Figure 4.

Spreading of cancer cells in a surrounding tissue depends on both cell migration and cell ability to invade surrounding tissues. Integrins directly support cell adhesion and migration [8]. For example, integrins αvβ3 and αvβ5 support cell adhesion to ECM through such proteins as osteopontin (SPP1, secreted phosphoprotein 1), periostin, fibronectin, which enables formation of tractions for migrating cells [9,49,50,51]. Several integrins (αvβ3, αvβ5, α3β1, α5β1, α6β1, α9β1) have been associated with the invasive phenotype of glioma cells [27,52,53,54,55]. Integrin-mediated signaling regulates activities and localization of proteases, which degrade ECM at an invasion front, including matrix metalloproteinases (MMPs), urokinase type plasminogen activator (uPA), as well as a disintegrin and metalloproteinase (ADAM). The expression of αvβ3 correlates with the levels of MMP-2 in invading glioma cells. Moreover, integrins promote the epithelial to mesenchymal transition (EMT), a process, in which epithelial cells reversibly acquire characteristics of invasive mesenchymal cells. EMT has been implicated in cancer invasion by providing a loss of cell–cell adhesion structures and increasing cell motility [56,57]. GBMs are not tumors of epithelial origin, however they adopt a phenotype that can be considered as mesenchymal and therefore the term EMT-like process or “glial to mesenchymal transition (GMT)” has been proposed [58,59]. α5β1 was shown to mediate this process in GBM cells [60].

### 4.2. Crosstalk with Growth Factor Receptors

Alterations in integrin signaling may also affect functions of other surface receptors [8]. Crosstalk between integrins and growth factors or cytokine receptors on both tumor and host cells is vital for many aspects of tumor progression [61,62]. In signaling via growth factor receptors, the ability of integrins to induce clustering of key enzymes and substrates may augment growth factor signaling, which is mediated by the same enzymes, including kinases and GTPases. Integrins can associate with and/or trigger cross-phosphorylation of a large number of RTKs (receptor tyrosine kinases), including epidermal growth factor receptor (EGFR), insulin-like growth factor receptor (IGFR) or vascular endothelial growth factor receptor (VEGFR). This process is mediated via recruitment of Src family kinases by activated FAK directly associating to integrins upon ligand binding.

Integrin-mediated adhesion to ECM can enhance growth factor signaling in yet another manner, i.e., by liberating growth factors bound to ECM proteoglycans [47]. Transforming growth factor (TGF) β is one of the growth factors associated with ECM proteins. In its inactive form, it is bound to and masked by a latency-associated peptide (LAP). Several αv integrins, mainly αvβ6 and αvβ8, can bind to the RGD motif within LAP (of TGF-β1 and TGF-β3) and cause an activation of TGF-β, which can subsequently bind to a specific receptor TGFβR and activate downstream signaling pathways [63]. In fact, lack of αvβ6 in mice remarkably phenocopied the defects detected in *TGF-β1* knock-out mice [64]. TGF-β1 enhances the cell surface expression of some integrins, i.e., β3, which promotes tumor migration. This interaction is important in remodeling of ECM by modulating the expression of genes encoding ECM proteins (fibronectin, laminin) and creating a proteolytic microenvironment (by up-regulation of MMPs and repression of inhibitors of these enzymes) [65,66,67]. In addition, TGF-β induces the expression of the transcription factor zinc-finger E-box binding homeobox 1 (ZEB1), which is involved in EMT transition in gliomas [68,69]. Moreover, TGF-β is involved in maintenance of cancer stem cell niche and acts as a potent immunosuppressive cytokine. Tumor cells expressing αvβ8 integrin evade host immunity by stimulating TGF-β signaling in immune cells [62]. Thus, integrin upregulation may activate multiple features of malignancy controlled by TGF-β including invasiveness, stemness and immunosuppression in human glioblastomas and other solid tumors [70,71].

### 4.3. Angiogenesis

Formation of new blood vessels in a tumor is crucial for its extensive growth and progression. Various integrins: α1β1, α2β1, αvβ3, α3β1, α5β1 that are expressed on endothelial cells promote angiogenesis by regulating migration and proliferation of endothelial cells [72,73,74,75]. Interestingly, deletion of αvβ3, αvβ5 and α5β1 in mice did not reduce angiogenesis in tumors formed by Lewis Lung carcinoma and B16-F10 melanoma cells [76]. This may indicate the compensatory effect of other integrin family members and demonstrates the complex interplay between various integrins in the regulation of angiogenesis. GBMs are among the most vascularized human tumors [77]. Newly formed endothelial cells, including those establishing tumor-associated vessels, predominantly express αvβ3 and αvβ5 integrins. Notably, the levels of αvβ3 and αvβ5 are relatively low in a quiescent endothelium, normal human brain and most adult epithelia [31,78]. Integrin α5β1 is induced on blood vessels, and its interaction with fibronectin supports angiogenesis [79]. Interactions of endothelial cells with surrounding ECM proteins, which are mediated by integrins, are important for vessel formation and maturation.

### 4.4. Glioma-Derived Integrin Ligands Interacting with Stromal Cells in GBM

Within a tumor microenvironment, functions of both resident stromal cells and infiltrating immune cells are modified by neoplastic cells. One of the main infiltrate in a tumor niche are tumor-associated macrophages (TAMs), which are the key responders to tumor-derived signals in many cancers. Instead of initiating anti-tumor responses, TAMs play an instrumental role in shaping the tumor niche by supporting tumor invasion, angiogenesis and by mobilization of different immunosuppressive cells from circulation and bone marrow [3,45,46,80]. Integrin receptors actively participate in regulating recruitment, trafficking and polarization of TAMs and other mobilized immune cells. Integrin α4β1 has been shown to promote colonization of tumors by myeloid cells in murine models of colon and lung cancers [81]. Antagonists of α4β1 inhibited the adhesion of monocytes to endothelium and their extravasation into tumor tissue from the circulation [81].

In malignant gliomas, tumor-derived factors attract brain resident microglia and peripheral macrophages, and reeducate them to perform similar functions to TAMs in peripheral cancers. We identified two αvβ3/αvβ5 integrin ligands, namely SPP1 and lactadherin, as glioma-derived factors responsible for polarization of glioma-associated microglia and macrophages (GAMs) into tumor-supporting cells [82]. SPP1 is a glycoprotein, which acts via integrin and CD44 receptors, and regulates adhesion, migration, invasion, chemotaxis and cell survival [83]. The levels of *SPP1* mRNA are up-regulated in human GBM and many other malignant cancer tissues. However, besides tumor cells, other cells within a tumor microenvironment express SPP1 [84,85]. We found up-regulated *Spp1* expression in glioma cells, but also in Cd11b+ cells (microglia and macrophages) in the rat and murine experimental gliomas [86]. Stromal astrocytes may also contribute to a pool of Spp1 in a tumor microenvironment. In a mouse model of PDGFB-driven glioma, *Spp1* was the most up-regulated gene in tumor-associated astrocytes of the perivascular niche as compared to normal brain astrocytes [87]. A competitive inhibitor of binding to αvβ3/αvβ5 integrin effectively blocked glioma-microglia interactions and microglia polarization *in vitro* [68]. Moreover, silencing of *Spp1* expression in glioma cells resulted in significant reduction of tumor growth and prevented polarization of GAMs in orthotopic syngeneic rat gliomas and in human glioma xenografts in immunodeficient mice [82] [our unpublished observation]. Likewise, Wei et al. showed that Spp1 via αvβ5 mediates a chemoattractive activity for recruitment and accumulation of GAMs in GBMs. Both tumor- and host-derived Spp1 were critical for glioma development, since ablation of *Spp1* from tumor cells or induction of tumors in *Spp1-/-*mice resulted in prolonged survival of tumor-bearing animals [88]. These apparently opposite activities of osteopontin in gliomas may stem from the fact that glioma-derived osteopontin is proteolytically degraded and is secreted as short fragments devoid of the pro-inflammatory activity [68].

The second identified glioma-derived glycoprotein, lactadherin (milk fat globule-epidermal growth factor 8, MFG-E8), enhances engulfment of apoptotic cells during phagocytosis. MGF-E8 acts by connecting phosphatidylserine on apoptotic cells and αvβ3/αvβ5-integrins on phagocytic cells [89,90]. Binding of MFG-E8 to αvβ3/αvβ5-integrin complexes on endothelial cells promotes VEGF-dependent neovascularization [91]. Activation of phagocytosis and angiogenesis supports tumor growth, however the role of MFG-E8 in cancer has been largely overlooked. Up-regulated MFG-E8 expression was reported in several types of cancers, including glioblastomas [92]. We demonstrated that *MFG-E8* knockdown in glioma cells reduces tumor growth and microglia and macrophage infiltration in orthotropic gliomas in rats [82].

Periostin is yet another glioma-derived protein, which interacts with integrins on microglia and macrophages in gliomas. Periostin is a multi-domain protein composed of a signal peptide (necessary for secretion), a small cysteine-rich motif (probably involved in formation of multimers through cysteine disulfide bonds), four fasciclin-like domains (FAS1) that bind with integrins (α_v_β3, α_v_β5, α_6_β4), and a hydrophilic C-terminal region known to interact with other ECM proteins such as collagens, fibronectin, tenascin C, or heparin. In murine gliomas, periostin was produced by glioma cancer stem cells (GSCs) residing in the perivascular niche and acted as a chemoattractant for brain macrophages through the integrin receptor αvβ3 [93]. Targeting these integrin receptors with the interfering RGD peptide (Arg-Gly-Asp-d-Phe-Lys) attenuated the interactions of microglia and macrophages with GSCs, reduced immune cell recruitment and their polarization into the tumor-supportive phenotype [93]. In another study, periostin gene silencing, using small interfering RNA, decreased TGF-β-induced expression of fibronectin and vimentin, partly through reduced Smad2, AKT and FAK phosphorylation, as well as invasion and migration of U-87 MG glioma cells [94].

The described examples of glioma–derived integrin ligands point clearly to an important role of αvβ3/αvβ5 integrin signaling in recruitment and induction of the pro-tumorigenic phenotype of microglia and macrophages associated with gliomas. Interestingly, in non-CNS (central nervous system) tumors, infiltration of macrophages was decreased in β3 integrin knockout (*Itgb3-/-*) mice, and resulted in the increased tumor burden in a colon cancer model [95]. In mice with deletion of the integrin β3 in macrophage-lineage cells, the increased growth of melanoma and breast cancers was observed and it was associated with the increased numbers of tumor-promoting macrophages along with decreased numbers of cytotoxic CD8+ T-cells [95].

Tenascin C (TNC) is an important integrin partner in stromal cells, which participates in many aspects of tumor progression. Tenascins are large glycoproteins, which are expressed during embryonic development, but their expression is limited in adult tissues. TNC are re-expressed under pathological conditions undergoing tissue/ECM remodeling, such as inflammation, tissue repair and cancer [96,97]. TNC is abundant in most types of solid tumors and its expression increases with the grade of malignancy [98]. Interestingly, the highest levels of TNC have been found in gliomas [99,100,101]. By binding to either integrin αvβ1 or αvβ6, TNC regulates cell adhesion and migration. Application of a peptide targeting TNC reduced the migration of glioma cells [102]. TNC also blocks a pro-survival signaling in endothelial cells through direct contact, but on the other hand it also induces a pro-angiogenic secretome in glioblastoma cells [103].

## 5. Interfering with Integrin Signaling as a Therapeutic Strategy against Glioblastoma

Since the discovery of the core integrin binding motives in laminin and fibronectin (YIGSR and RGD, respectively) in 1980s/1990s, these sequences attracted a lot of attention in search for anticancer therapies. Early studies showed that blocking a large subset of integrins, including α5β1, αvβ3, αvβ5, using peptides containing an RGD motif interferes with the invasion of tumor cells *in vitro* and formation of metastasis in murine cancer models. Interestingly, peptides with the RGD motif inhibited also tumor angiogenesis. Based on these initial promising findings, a variety of RGD-containing peptides, peptides with alternative integrin recognition motifs and mimetics have been developed [8].

A cyclic pentapeptide blocking the RGD binding site called cilengitide (EMD 121974, cyclo-Arg-Gly-Asp-DPhe-NMe-Val) was identified as a potent and selective αvβ3 and αvβ5 integrin antagonist. It displayed greater stability and antagonistic activity than its linear counterpart [104]. In pre-clinical studies, cilengitide showed bimodal action on blood vessels and brain tumor cells, by inducing cell death (anoikis, a cell death due to a lack of anchorage) in the angiogenic endothelium and glioma cells *in vitro* [105]. Its anti-tumoral effects *in vivo* were dependent on anti-angiogenic, cytotoxic and anti-invasive activities [106]. Cilengitide effectively inhibited the growth of orthotopic gliomas and, importantly, brain microenvironment was a crucial determinant of the susceptibility of these tumors to cilengitide. Tumors implanted to the flank of the same mice were unaffected by the treatment with this drug [107].

Cilengitide has been the first, and so far the most advanced, among various integrin-targeting RGD pentapeptides tested in clinical studies. Cilengitide was effective without significant toxicity both as a single agent and when combined with radio- and chemotherapy in GBM patients in the phase I/IIa studies [108,109]. In the phase II clinical trial, positive effects of combination of cilengitide with temozolomide (a standard chemotherapeutic in GBM) was observed in patients with the *MGMT* gene promotor methylated. The *MGMT* gene encodes O6-methylguanine DNA methyltransferase, known as a predictive marker for the success of temozolomide therapy. However, recently published results of the randomized phase III CENTRIC and phase II CORE clinical trials, which investigated the efficacy of cilengitide in newly diagnosed GBMs, in combination with standard radiotherapy plus temozolomide, did not show consistent effects on overall survival and progression-free survival [110,111]. The reasons for the failure of cilengitide in the clinic could be due to: its short half-life, quick clearance from circulation, inefficient penetration across BBB and subsequent low brain accumulation, and quick clearance from tumor tissues, which prevented from reaching therapeutic levels in tumors [112]. Importantly, previous studies showed that the treatment with cilengitide at low-doses actually enhanced VEGF-mediated angiogenesis and tumor growth [113]. Recently, Zhao and co-workers proposed to tackle quick clearance issues with nanotechnology and overcome BBB by combining nanotherapy with sonoporation. After encapsulation of cilengitide in heparin–poloxamer based nanoparticles, the drug was efficiently delivered across BBB and accumulated in the tumor in a rat glioma model. Meanwhile, its distribution to non-targeted organs was reduced [114]. Reconsidering the use of cilengitide or any other RGD-based treatments will possibly require a more careful selection of a biomarker(s) to identify patients, who would be better responders and benefit more from the treatment. Integrins αvβ3, αvβ5 and αvβ8 are differentially expressed in GBMs. In the retrospective study on the CORE trial cohort of patients, higher αvβ3 levels in tumor cells (but not in endothelial cells), and not αvβ5 levels, were associated with the improved outcome in patients treated with cilengitide [30].

High affinity interaction of the α5β1 integrin with fibronectin requires both the RGD sequence and a second recognition motif, which synergizes with RGD to strengthen the binding [8]. ATN-161 (Ac-PHSCN-NH2; volociximab), a 5–amino acid peptide, was derived from this so-called “synergy sequence” PHSRN of fibronectin. ATN-161 was shown to inhibit tumor growth, angiogenesis and metastasis, and extend survival in several animal tumor models, including breast, colon and prostate cancer, either when given as a single agent or when combined with chemotherapy and radiotherapy [115,116]. A phase I clinical trial showed a very good safety profile for the use of ATN-161 in patients with advanced solid tumors [117]. Unfortunately, due to low activity of ATN-161 in the phase-I/II for recurrent gliomas and the phase-II for renal cancer in USA studies were halted in February 2016 (http://adisinsight.springer.com/drugs/800018411).

Despite discouraging results, inhibition of integrins as a potential therapeutic strategy in GBM has not been abandoned. It even prompted efforts to develop new peptidic and non-peptidic integrin antagonists with different scaffolds and a different pattern of binding properties, however those studies still concentrate mainly on antagonism with RGD binding integrins. Several of these compounds showed promising results against GBM cells *in vitro* (for review [118]). A new player in the treatment of GBM is GLPG0187, a small molecule integrin antagonist. This compound blocks a broad spectrum of RGD integrin receptors (αvβ1, αvβ3, αvβ5, αvβ6, αvβ8, α5β1). GLPG0187 showed anti-tumor activity in preclinical models of breast and prostate cancer [119], as well as effectiveness against glioma cells [66]. Preliminary results of the phase Ib study confirmed a favorable toxicity profile of the drug and early signs of clinical response in patients with progressive high-grade glioma and other solid tumors [120].

Beyond inhibition of integrin function, another way of clinical exploitation of the increased levels of integrins in gliomas is their use for targeted delivery of radiotherapy, chemotherapy or gene therapy agents. ^60^Y-labeled Abergin, a humanized monoclonal antibody against human integrin αvβ3, was used in integrin-targeting radioimmunotherapy and showed reduction of the tumor volume in the orthotopic GBM model [121]. Nanoparticle vehicles conjugated to an integrin binding peptide (e.g., RGDK) can transport a variety of cargoes, such as small cytotoxic molecules and biomolecules (plasmid DNA, siRNA, oligonucleotides, peptides) to a desired tumor site [122]. Specific pattern of integrin expression in glioma is also used for diagnostic applications. Radiolabeled or fluorescent RGD-based probes allow for precise GBM localization via αvβ3 imaging [24,123].

## 6. Conclusions and Perspectives

Integrins mediate a crosstalk between cells, ECM, growth factor receptors and mechanical stimuli. They are involved in many processes associated with diffusive and invasive growth, extensive vascularization and increased migratory potential in pseudopalisading hypoxic regions adjacent to necrotic foci that are major hallmarks of glioblastoma. Despite promising results of blocking of a single integrin *in vitro* and evidence of its importance for cancer progression in the preclinical experiments, a therapeutic use of a selective integrin antagonist has been unsuccessful in clinical settings. Identification of a full spectrum of integrins that are expressed on a variety of cells within the TME and understanding their combined roles is critical for effective exploitation of integrins as anti-cancer targets.

Noteworthy, glioma cells are capable of migrating not just perivascularly in an integrin-dependent manner, but also down the white matters tracts using CD44 proteins (cluster designation 44; HCAM, homing-associated cell adhesion molecule). CD44 is a transmembrane receptor for a HA-rich environment [124]. Both integrins and CD44 have been extensively studied in cancer and play a significant cooperative role in the invasive behavior of malignant gliomas. Inhibition of integrins may be thus insufficient to prevent an invasive growth of GBMs due to the presence of redundant pathways or hijacking some cytoprotective mechanisms by cancer cells. CD44 expression is upregulated in GBMs and varies in different glioma subtypes, with lower levels expressed in the pro-neural subtype, the highest in the mesenchymal subtype and intermediate levels in the classical subtype [39]. In addition, CD44 has also been shown to promote the GBM stem-like phenotype via SPP1, a dual integrin and CD44 ligand [125,126]. Thus, integrins and CD44 cooperate to support tumor cell migration/invasion and are key regulators in maintaining stemness of glioma stem-like cells [54]. Combining CD44 and integrin antagonists may potentially produce a more effective treatment [39].

Implementation of genetic analysis to the new WHO classification of brain tumors provides more precise diagnosis for adjustment of therapy regiments. Hopefully, molecular evaluation of GBMs would soon serve for diagnostic, prognostic or epidemiologic purposes, as well as for designing personalized treatment with appreciation of full complexity of this heterogenous, incurable disease.
